# Case of Tic Douloureux, Successfully Treated by Division of the Affected Nerve

**Published:** 1820-05

**Authors:** 


FOR THE LONDON MEDICAL AND PHYSICAL JOURNAL.

*Case of Tic Douloureux, successfully treated by Division of the affected Nerve.* By Professor Richerand.

PILLEUX, forty-eight years of age, of tall stature, and a dry and nervous constitution, had been much exposed to severe vicissitudes of weather during a military life of twenty-eight years' duration. On the 24th of August, 1819, he first suffered all the symptoms of maxillo-dentary neuralgia on the left side. The pain arose from the point where the maxillo-dentary nerve penetrates the interior of the inferior maxillary bone; it occupied the whole left side of the body of this bone, and passed out (if the expression may be admitted) at the foramen at the chin; it thence ran up the cheek, and extended through all the ramifications of the maxillary branch of the trifacial nerve. The patient said it seemed as if the left side of his face was traversed by a red-hot iron. During the time that this intense pain existed, the lower lip and all the left side of the face trembled, palpitated, and was as it were agitated by a sort of vibratory movement. The saliva flowed from the mouth in abundance, and the left eye was dim. Several attacks of this kind came on in the night of the 24th of August. During the eight following days, the paroxysms occurred twenty-five or thirty times every twenty-four hours: the interval was sometimes not above five minutes, and the pain continued sometimes for nearly half-an-hour. All the affected side of the face remained swelled. On attempting to speak or to masticate, a paroxysm was immediately produced. The patient was deprived of sleep for six weeks. Moxa had been twice applied on the lower jaw. The frequency of the fits then diminished; but, as the patient could not procure, whilst at home, the means proper for the cure of his malady, he entered the Hospital Saint-Louis on the 7th of October. For seven days after this he suffered no pain; but the paroxysms of neuralgia recurred in rapid succession on the 13th, and were more violent than the former ones.

*Treatment.*—From his entry into the hospital until the 1st of December, gargles either of opium, decoction of pyrethrum, sal ammoniac and alcohol, or muriate of potash mingled with powder of liquorice, were used; an anodyne cataplasm was kept constantly applied over the face; and opium, with purgatives, were exhibited internally. These measures were not devoid of utility; for, during the last fifteen days in November, the patient suffered only one attack in twenty-four, and sometimes even in forty-eight, hours. But he was so anxious to get entire relief, that he readily submitted to the operation of the division of the affected nerve, which had been proposed to him. The section of the nerve was made just as it passed out of the foramen at the chin, by a single stroke of the bistoury. The patient has experienced no pain since that time. The side of the lip corresponding to the divided nerve remained in a state of numbness for several days; but the sensibility of it returned after this, and the patient left the hospital on the 28th of December (twenty-one days after the operation), perfectly well.

